# Double life: How GRK2 and β-arrestin signaling participate in diseases

**DOI:** 10.1016/j.cellsig.2022.110333

**Published:** 2022-04-14

**Authors:** Ruxu Zhai, Jonathan Snyder, Sarah Montgomery, Priscila Y. Sato

**Affiliations:** Drexel University College of Medicine, Department of Pharmacology and Physiology, Philadelphia, PA, USA

**Keywords:** GPCR regulation, arrestins, GRK, diseases, metabolism, cancer, pain, heart failure

## Abstract

G-protein coupled receptor (GPCR) kinases (GRKs) and β-arrestins play key roles in GPCR and non-GPCR cellular responses. In fact, GRKs and arrestins are involved in a plethora of pathways vital for physiological maintenance of inter- and intracellular communication. Here we review decades of research literature spanning from the discovery, identification of key structural elements, and findings supporting the diverse roles of these proteins in GPCR-mediated pathways. We then describe how GRK2 and β-arrestins partake in non-GPCR signaling and briefly summarize their involvement in various pathologies. We conclude by presenting gaps in knowledge and our prospective on the promising pharmacological potential in targeting these proteins and/or downstream signaling. Future research is warranted and paramount for untangling these novel and promising roles for GRK2 and arrestins in metabolism and disease progression.

## 1. GPCR signaling and receptor barcoding

G protein-coupled receptors (GPCRs) are seven transmembrane domain proteins that upon agonist binding activate several downstream pathways involved in various cellular processes. GPCRs compose one of the largest protein superfamilies which importantly account for the targets of over 35% of FDA-approved pharmacological agents [1]. Importantly, a vast number of GPCRs are orphaned receptors, that is, an agonist and/or function remains unknown. Thus, there is a dire ongoing need to better understand GPCR-mediated mechanisms in the context of both disease and pharmacological intervention and to unravel novel GPCRs and GPCR-regulating proteins. The extensive involvement of this receptor family and its regulators in various pathologies (section 4) highlights the impact of GPCR-pathways in regulating cellular function and in many cases driving pathological signaling.

GPCRs can be classified into seven main subfamilies. Class A (Rhodopsin-like receptor family) is the largest GPCR subfamily which includes adrenergic receptors. Class B (Secretin receptor family) comprises peptide hormones like glucagon-like peptide-1 receptor (GLP1R) [2]. Class C (Metabotropic glutamate receptor family) consists of glutamate and GABA_B_ receptors that form special allosteric dimers [3]. Class D includes fungal mating pheromone receptors. Class E notably includes cAMP receptors; Class F (Frizzled (FZD) / Taste receptor family) have been described for their role in regulating transcription, cytoskeleton, and calcium [4–6]; and lastly the adhesion receptor family [7,8].

**Fig. 1.**
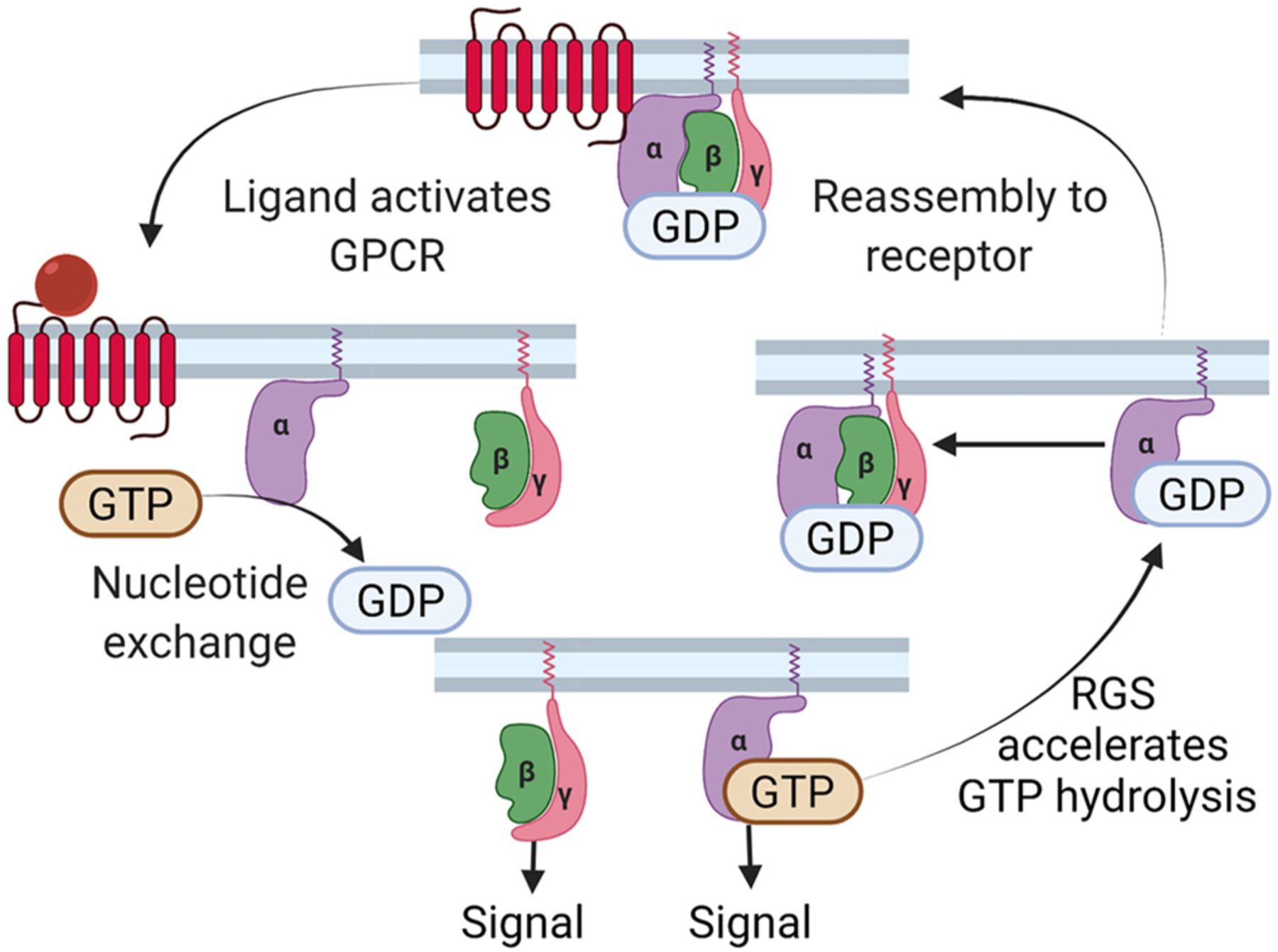
Schematic representation of GPCR-G-protein signaling. Created with BioRender.

GPCR signaling patterns are principally determined by the binding of ligands in the extracellular domain and the interaction with cytosolic signaling partners on the intracellular interface (Fig. 1). Each GPCR has a unique binding profile for one or more ligands, each of which with specific receptor interaction dynamics. This review will focus on the important regulatory events in the intracellular side of GPCR activation which controls its localization and capacity to induce second messenger signaling cascades. The unique signaling pattern of a GPCR is informed by the specific heterotrimeric G-protein complex to which it is coupled [9]. The intracellular guanine nucleotide heterotrimeric G protein complex that couples the GPCR includes Gα, Gβ, and Gγ subunits [10] (Fig. 1). The Gα subunit can be further characterized into four sub-families, Gαs, Gαi/o, Gαq/11, and Gα12/13, based on structure similarity and activity [11]. The human proteome contains 23 Gα, 5 Gβ, and 12 Gγ [12], which theoretically could form more than 1300 heterotrimeric complexes giving GPCRs a vast ability to fine-tune signaling cascades based on specific heterotrimeric G-protein coupling. Upon stimulation, the active-inactive cycle of GPCR begins with the dissociation of guanosine diphosphate (GDP, inactive form) from the Gα subunit which allows for guanosine triphosphate (GTP, active form) to bind [13–15]. The Gα subunit then dissociates from the Gβγ dimer and activates second messengers including the adenylyl cyclase (AC)/cyclic AMP (cAMP) catalyzation cascade, inositol triphosphate (IP3), and/or ion channels. G-protein signaling is terminated by GTP hydrolysis catalyzed by regulator of G protein signaling (RGS) proteins. This allows G-proteins to return to their unstimulated GPCR-bound state [16] (Fig. 1). Moreover, coupling of specific GPCRs to G-protein complexes can be cell type-specific. For instance, D1-like dopamine receptors canonically couple to Gαs while D2-like dopamine receptors canonically couple to Gαi; however, in certain brain regions and in macrophages it has been documented that both classes can primarily couple to Gαq [17,18]. This allows for an incredible number of GPCR-G protein coding and integration permitting a very finely-tune signaling pattern that may differ in specific cell types as well as allowing for different cellular responses to the same stimuli.

**Fig. 2.**
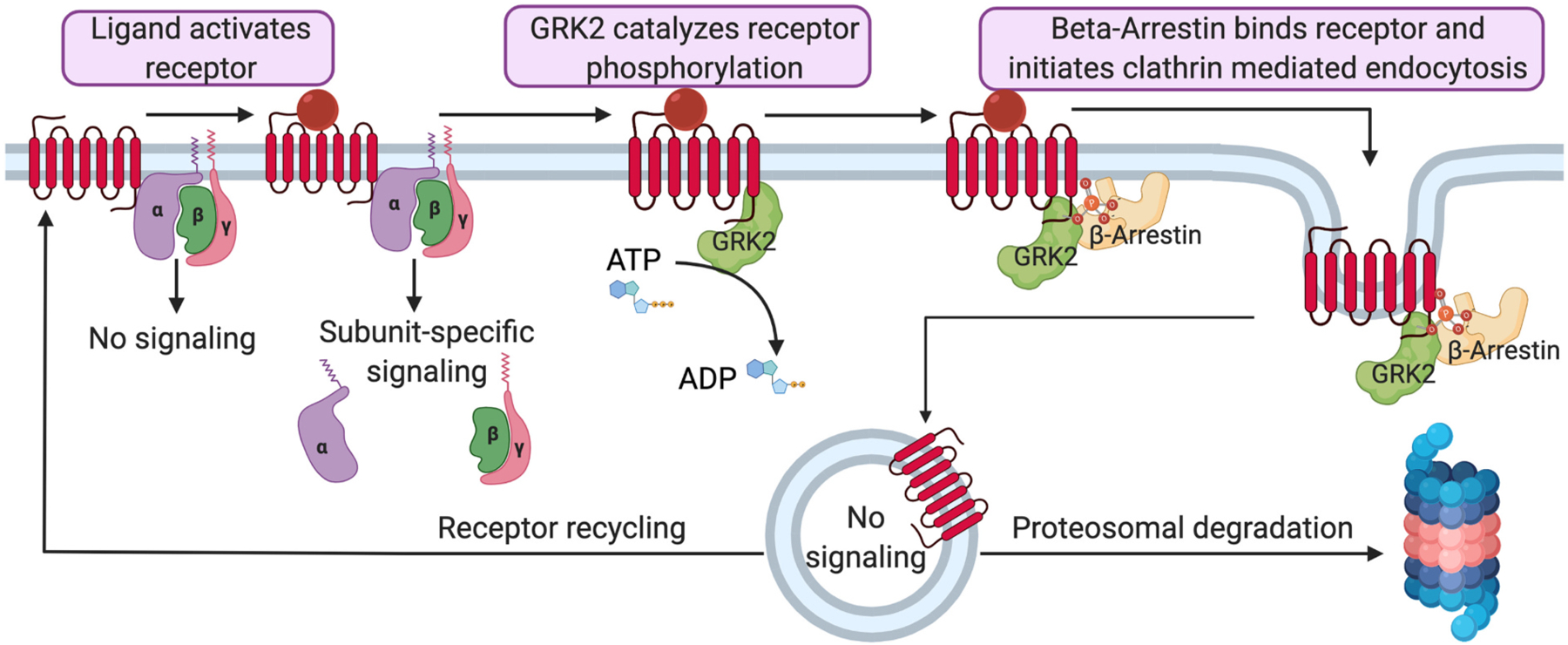
GRK2 and β-arrestin signaling in receptor internalization. Created with BioRender.

Notably, these cell type and cell state deviations in signaling have also been proposed to be associated with post-translational modifications to the GPCR’s intracellular surface. An important and increasingly well-characterized domain of post-translational modification to GPCRs is referred to as “GPCR barcoding”. GPCR barcoding refers to the multitude of regulatory phosphorylation and dephosphorylation events mediated by kinases and phosphatases on the intracellular side of an activated GPCR. These phosphorylation patterns inform the energetic favorability for binding of intracellular proteins. For instance, a rapid turn-off mechanism can be accomplished by GPCR-kinase (GRK) receptor phosphorylation which promotes binding of β-arrestins [19,20]. The recruitment of β-arrestins allows for clathrin-mediated endocytic internalization of the receptor for recycling and/or degradation (Fig. 2). This review will focus on the role of GRKs and β-arrestins in cellular function and diseased conditions. Noteworthy, other kinases are well known to participate in receptor barcoding such as Protein Kinase A (PKA) [21] and Protein Kinase C (PKC) [22]. For instance, increased PKA-mediated phosphorylation of the β2-adrenergic receptor (β2AR) can direct its signaling from Gαs to Gαi [23]. These regulatory phosphorylation events can be classified as second messenger dependent (PKA and PKC) or as ligand binding dependent (GRK family). Finally, it is important to note that, while these two classes of kinases both regulate GPCR signaling through barcoding, they can also directly interact with each other, further integrating second messenger-dependent and ligand-dependent GPCR barcoding. For instance, PKA is known to phosphorylate GRK2 and thereby enhance its desensitization of the β2-adrenergic receptors and potentially other receptors [24]. However, their contributions to the GPCR barcode remain distinguishable as PKA phosphorylation sites do not appear to influence β-arrestin recruitment, whereas GRKs are the primary driving force towards this GPCR fate [25].

### 1.1. GRK Family

Phosphorylation of activated GPCRs by GRKs was first described almost five decades ago where rhodopsin, a classical GPCR, was found to be phosphorylated by rhodopsin kinase, now known as GRK1 [26]. The β2AR was subsequently found to be phosphorylated and desensitized by β-adrenergic receptor kinase 1 (also known as GRK2), and found to be altered in various pathologies [27]. Several additional GRK isoforms have since been identified and they are now divided into three main groups: (i) the rhodopsin kinase subfamily which includes GRK1 and GRK7; (ii) the β-adrenergic receptor kinase subfamily, which includes GRK2, and GRK3; and (iii) the GRK4 subfamily includes GRK4, GRK5, and GRK6 [28]. GRK1 is expressed in rods (low/dim light) and cones (bright light with color distinction) and can inactivate vertebrate photoreceptors and regulate the phototransduction cascade. GRK7 is expressed primarily in cones and regulates the same processes [29]. GRK3, also known as β-adrenergic receptor kinase 2, is the least abundant cytosolic GRK, but it is widely expressed in the brain [30]. GRK4 plays an important role in the phosphorylation and regulation of dopamine D1 receptors, and when overexpressed induces hypertension [31]; although, GRK4 is mainly expressed in the testis, cerebellum, and kidneys [32]. GRK4 and GRK6 are post-translationally modified to localize to the membrane [33]. GRK2 and GRK5 are unevenly expressed in different tissues throughout the body and are well characterized for their roles in heart function [34]. Notably, GRK5 is associated with the membrane through PIP2 and phospholipid binding [35]. Due to its broad expression pattern, potential for pharmacological targeting, and involvement in a spectrum of diseases, this review will focus on GRK2.

### 1.2. Molecular determinants of GRK2 signaling

GRK family members have similar structures including a conserved central catalytic domain, an N-terminal domain that recognizes and localizes the kinases to lipid membranes, and the variable C-terminal domain which regulates their translocation [36,37]. However, there are three distinct regions in GRK2 that play key roles in determining its function: an amino terminal RGS homology (RH) domain, a pleckstrin homology (PH) domain, and a unique C-terminal domain. The RH domain of GRK2 [38] has been shown to specifically interact with members of the Gαq and Gα11 family and is able to inhibit Gαq-mediated phospholipase C activity [39]. The PH domain participates in its translocation to the plasma membrane as it is implicated in binding phospholipids and Gβγ subunits. Table 1 shows a subset of known GPCRs with their respective primary functions that are negatively regulated by GRK2-β-arrestin interactions. Thus, upregulation of GRK2 or β-arrestin activity by natural or pharmacological agents would detrimentally impact receptor activity while the reverse would enhance its receptor primary responses.

GRK2 induces the desensitization of GPCRs through multiple phosphorylation events at unique sites specific to each GPCR. Distinguishing which phosphorylation sites are specific to GRK2 relative to other GRKs or second messenger-dependent protein kinases requires using multiple site-specific mutations, functional analysis, and well validated phospho-specific antibodies. Such studies have been performed for some key GRK2-regulated receptors including the β2AR, where GRK2 phosphorylates threonine 360 and serine residues 364, 396, 401, 407, and 411 within the C-terminal tail [52]. Conversely, in dopamine receptors, since specific sites are not well established, the GPCR-GRK2 interaction is studied by mutating all intracellular serine and threonine residues to alanine [53,54]. Some studies have attempted to predict GRK2 phosphorylation sites by computational methods using available crystal structures of GRK2 and its interacting partners, considering residue accessibility and structural flexibility [55]. While much has been detailed regarding certain subtypes of GPCRs and its GRK2 interactions, many of GRK2’s preference for specific GPCRs remain to be further detailed.

### 1.3. The complexity of GRK2 signaling beyond GPCR phosphorylation

In addition to regulating processes via phosphorylation, GRK2 can regulate cellular responses in a non-phosphorylation-dependent manner, such as through structural interactions with proteins involved in signal transduction and transport. This includes facilitating the recruitment of phosphoinositide 3-kinase (PI3K) to the membrane and aiding in receptor desensitization [56]. The binding of serine-threonine kinase Akt through the C-terminus of GRK2 can result in inhibiting phosphorylation of Akt [57]. The role of upregulated GRK2 on decreasing extracellular signal-regulated kinase (ERK) activity is independent of receptor phosphorylation, in a proposed mechanism involving direct or coordinate downstream interaction with the chemokine-driven mitogen-activated protein kinase (MEK) [58]. The interaction between Raf and MEK could be dissociated by the Raf kinase inhibitor protein (RKIP), which was also shown to activate GRK2, and further inhibits the kinase signaling through the Ras-Raf-MEK-ERK pathway [59]. Clathrin binds to GRK2 through a clathrin box located in the C-terminal domain of GRK2 and induces the internalization of certain GPCRs through β-arrestin recruitment [58,60]. Protein-coupled receptor kinase-interacting protein (GIT1), a GTPase-activating protein (GAP), was shown to interact with GRK2 [61]. The inhibitory binding of calmodulin to GRK2 could be almost completely abolished by PKC phosphorylation at serine 29, and resulted in decreased kinase activity of GRK2 [62]. GRK2 also associates with caveolin, α-actinin, proto-oncogene tyrosine-protein kinase Src (c-SRC), IkBα, and heat shock protein 90 (Hsp90), a folding regulator of GRK2 [36,63–65]. Notably, GRK2 phosphorylates non–GPCR proteins, such as the insulin receptor substrate 1 (IRS1), an important regulator of insulin sensitivity and glucose uptake [66]. Inhibition of GRK2 enhanced endothelial nitric oxide synthase (eNOS) signaling, which reduced myocyte loss and improved ischemic injury in mice [67]. Thus, GRK2 is an important regulator of cell death, cell-cell interaction, cytoskeletal networks through tubulin and actin [68], immunity and other intracellular functions via mechanisms that also involve its non-GPCR non-kinase function.

Just as receptor phosphorylation by GRKs can trigger internalization and desensitization, residue specific dephosphorylation can oppose this process and promote re-sensitization. Receptors internalized by endocytosis must undergo dephosphorylation to be meaningfully recycled to the plasma membrane (Fig. 2) as a GPCR barcoded for internalization would be short-lived at the plasma membrane [69]. Dephosphorylation may also occur at the plasma membrane prior to endocytic engulfment, thus preventing internalization [70]. As with the GRK family of kinases, phosphatases that regulate this process do not solely interact with GPCRs [71] and they possess functional redundancy (e.g. multiple phosphatases are able to regulate one receptor). Delineating what causes a receptor to be recycled or degraded is of great importance. For instance, a therapeutic strategy that favored central nervous system opioid receptor recycling over degradation has been sought after for some time [72]. In addition to phosphatase activity, ubiquitination and de-ubiquitination can contribute to this process as an ubiquitin-tagged protein will be targeted for degradation [73]. As such, the details of how GPCR post-internalization sorting occurs merits its own review [74].

## 2. Arrestins in Cellular Signaling

β-Arrestins are a family of regulatory proteins that recognize and bind to activated GPCRs with high affinity to promote receptor desensitization, turning off continued signaling [75,76]. The visual arrestin-1 (also known as S-Arrestin) was first described in 1986 as a 48-kDa protein that phosphorylates and suppresses rhodopsin’s cGMP phosphodiesterase activating capacity [76]. A second arrestin was then found to bind competitively with phosphorylated rhodopsin and phosphorylated β2AR. For this non-visual interaction, it garnered the name β-arrestin; it was later termed β-arrestin1 following the discovery of the second non-visual (third in total) arrestin. The third arrestin homolog (β-arrestin 2) was isolated from bovine brain cDNAs [77,78]. β-arrestin 1 and 2 have more than 70% conserved amino acid identity and considerable functional overlap, yet with some functional distinctions [79,80]. Despite being named for their initial association with the β2AR, it is now known that β-arrestins interact with more than 100 proteins and many other GPCRs [81]. A fourth arrestin, a visual arrestin with restricted expression in photoreceptors cells, was later reported where cDNA was isolated from cone photoreceptor of ascidian Ciona intestinalis [82]. Focus of this review will be on non-visual β-arrestin 1 and β-arrestin 2 as it relates to GRK signaling and diseases.

### 2.1. Molecular determinants of β-arrestin signaling

Crystal structures for β-arrestin 1 [83] and for β-arrestin 1 and 2 in complex with a number of GPCRs [84] or GPCR fragments [85] have revealed key structural determinants of these proteins. β-arrestins have two separate noteworthy binding domains, where one domain recognizes phosphorylation sites on the C-terminal tail of GPCRs and a second domain recognizes whether the receptor is in an active conformation. These two domains have been proposed to induce an active state of β-arrestin [86], where both separate binding events are thought to contribute to β-arrestin activity and GPCR desensitization. Upon binding an activated and phosphorylated GPCR, β-arrestin undergoes a conformational change that rearranges both its C- and N-termini exposing a third critical domain, the clathrin-binding domain. The exposure of this domain in its active form initiates the formation of the clathrin-coated endocytic vesicle [87], which is key to receptor desensitization processes.

### 2.2. β-arrestin in biased and unbiased signaling

In the standard model of GPCR signaling, agonist binding induces signaling through the heterotrimeric G-protein complex equivalently, and thus one agonist binding event will in equal measure induce second messenger signaling and receptor desensitization. However, many GPCRs have multiple natural and synthetic agonists, some of which can favor the induction of signaling or desensitization of the receptor relative to the primary cognate agonist, through a process known as biased agonism. The model for biased agonism is based on the principle that GPCRs exhibit ligand-dependent conformational changes resulting in ligand-dependent signal transduction [88]. For instance, there are a number of biased agonists for the β2AR either towards greater G-protein signaling or greater desensitization each with distinct clinical utility which was recently reviewed by Ippolito and colleagues [89]. For instance, inhaled β2AR agonists biased towards G-protein signaling have greater utility in asthma than desensitization-biased agonists. GRKs and arrestins binding to an activated GPCR is a key determinant of whether an agonist will exhibit this biased effect. As mentioned above, bias is driven by ligand-dependent conformational changes, and indeed some ligands induce conformational changes that energetically favor GRK2 binding. This is well demonstrated by FRET and BRET studies showing real time differential GRK2 [90] and/or β-arrestin colocalization to GPCRs in response to biased and non-biased ligands [91]. From a functional standpoint, it has been demonstrated that by prohibiting phosphorylation by GRKs through site-specific mutagenesis, a non-biased agonist can exhibit the effects of a G-protein biased agonist [92]. Exploring signaling one way or another and determining key events leading to β-arrestin biased signaling may be pivotal for developing targeted pharmacological agents to treat specific pathologies.

## 3. GRK2 and β-arrestin signaling

β-arrestins can sterically limit the attachment of G-protein heterotrimers with the receptor and therefore prevent its reassembly with the receptor. GPCR phosphorylation by GRK2 favors the recruitment of β-arrestin and in turn, induce receptors desensitization, promoting rapid receptor internalization [93]. Other subtypes of GRKs involved in desensitization and β-arrestin recruitment have different efficiencies. For instance, GRK2 and GRK3 are more effective in promoting receptor endocytosis through the β-arrestin pathway than GRK5 and GRK6 [25]. New studies have proposed key events and cross-talk between GRK2 and arrestins of which we summarize in the following subsections.

### 3.1. Non-GPCR signaling of GRK2/β-arrestin

In addition to the inhibitory effect of GRK2/arrestins in GPCR signal transduction, emerging evidence suggests that GRK2 and β-arrestins regulate intracellular signaling independently of GPCRs by affecting non-GPCR receptors or by direct interaction with various cellular target molecules involved in signal transduction. β-arrestins can be used as scaffolds to bring GPCR-GRK2 complexes in close proximity with different signal molecules, including JNK-3, IGF-1R, and IGF-1, PDE4, cytoskeleton Ral-GDS modulator, Nuclear factor-κB (NF-κB) signal pathway, Mdm2, etc [94–96]. GRK2 and β-arrestins can be localized in other cellular compartments (i.e. mitochondria) where binding partners and cellular functional impact remain to be fully elucidated. Thus, GRK2 interaction with β-arrestins is essential for “turning-off” GPCRs but may also be key to overall cellular signal cascade that depend or not of GPCR responses.

### 3.2. GRK2/β-arrestin in metabolic regulation

Mitochondrial localization of GRK2 was first evidenced in rat cerebrovascular tissue and has been proposed to contribute to the progression of Alzheimer’s disease (AD) [97]. The kinase role of GRK2 in the mitochondria was then reported to regulate biological processes and ATP generation in primary cultured mouse aorta cells [98]. Recently, emerging evidence supported the metabolic regulatory role for the non-canonically mitochondrial localized GRK2 where it participates in cellular metabolism post cardiac ischemia reperfusion injury [99]. Supporting data suggests that indeed GRK2 translocates to the mitochondria via ERK phosphorylation of GRK2 and subsequent interaction of GRK2 with Hsp90 [100] where it inhibits glucose oxidation post-IR via decreased pyruvate dehydrogenase activity [99]. Although functionally mitochondrial GRK2 has been shown to negatively impact metabolic function and promote ROS-formation [101] clearly defined mitoGRK2-protein interactions within the mitochondria remains to be fully detailed. These interactions could be important to regulating mitochondrial substrate utilization particularly in diseased conditions such as myocardial infarction.

In addition to critically regulating metabolic processes, mitochondria can induce cell death responses to stress by releasing cytochrome c into the cytosol and promoting caspase activation [102]. Interestingly, GRK2 has been shown to induce cell death by increasing cleaved caspase 3 post-IR, whereas preventing its translocation leads to decreased CC3 and cell death post-IR [100,103]. Recent studies have also shown that apoptosis-induced caspases cleave β-arrestin 2 which then translocates to mitochondria to increase the release of cytochrome C into the cytosol, enhancing apoptotic signaling [104]. In addition, β-arrestin 1 mediates the activation of PI3K, subsequently activating Akt-induced apoptosis [105]. GRK2 and β-arrestins have been proposed to regulate mitochondrial function in cardiac cells through mechanisms involving mitochondrial superoxide production via NADPH oxidase 4 (Nox4) [106,107]. Noteworthy, β-arrestins have been shown to affect several proteins involved in mitochondrial respiration and metabolism like GAPDH in the glycolysis pathway and ATP synthases [81]. Exact mechanisms of action are not fully known albeit necessary to further understand the role of these proteins in metabolic regulation.

### 3.3. Beta-arrestin as a transcription factor

β-arrestins regulate gene transcription in the nucleus through cytosolic and nuclear interactions. β-arrestins bind to IkB and sequester the complex IkB-NF-κB in the cytosol, thus inhibiting NF-κB transcriptional activity, critical for inflammasome priming [108,109]. Although the ability of β-arrestin 1 to alter cellular transcription was well accepted, whether β-arrestin exerted this function via direct nuclear interactions was largely debated until an active nuclear localization signal was identified in β-arrestin 1 [110]. Since then, several studies have reported β-arrestin 1 as an epigenetic regulator in various cell types [111–113]. Importantly, β-arrestin 1 localization in the nucleus was dependent on GPCR activation [110] suggesting that its nuclear activity may be dependent on GRK initiation of GPCR-β-Arrestin interactions and scaffolding complexes. Further studies are needed to clarify these mechanisms.

### 3.4. Mutations and differential expression of GRKs and β-arrestin in animal disease models

Using targeted knockout and heterozygous mice, GRK2 has been shown to play an important role in atherosclerosis and other heart diseases [114–116], lymphocyte chemotaxis [117], and autoimmune diseases [118]. There are various genetic and chemically induced mouse models utilized to study GRK2/β-arrestin signaling. Studies have shown that cardiac overexpression of GRK2, as evidenced in human heart failure, is detrimental to myocardial function, particularly post-cardiac injury or pathological hypertrophy [119]. Global small molecule GRK2 inhibition is available through several compounds (including paroxetine, compound 101, balonal, etc), each posing a unique degree of selectivity and potency [120]. Alternatively, tissue-specific inhibition has been accomplished using expression of the small peptide termed βARKct [121].

GRK2 knockout strategies were performed by targeting exon 8, where homozygous GRK2 knockout mice were embryonically lethal [122]. Particularly, a lethality was linked to abnormal cardiac development, possibly due to markedly hypoplastic ventricular myocardium with abnormally lacking ventricular myocardium organization or differentiation during early embryogenesis, leading to heart failure. This mouse model supported the notion that GRK2 plays a critical role in development, particularly of the heart [122]. Interestingly, the global heterozygous GRK2 knockout mice were viable, indicating a minimal protein expression level that reached threshold for successful cellular differentiation and development. Cardiac-specific GRK2 knockout mice were generated using the Cre-lox technology, where mice were viable with normal heart structure and function in early development. Nonetheless, β-adrenergic stimulation revealed increased sensitivity of inotropic and lusitropic tachyphylaxis in the adult heart, suggesting the importance of cardiac GRK2 in protective effects in cardiomyopathy induced by catecholamine toxicity [115]. Further studies, using pancreatic-specific GRK2 knockout mice revealed a decrease in glucose-mediated insulin secretion linked to decreased calcium influx in pancreatic islets [123]. Whether this is developmentally linked remains to be further investigated but may be an important contributor to diabetes disease progression.

Murine knockout of β-arrestin 1 were generated by genetic disruption via homologous recombination at mouse chromosome 7. The study showed a similar survival rate of the β-arrestin 1 KO mice with wild-type mice, accompanied with similar fertile and offspring generation. Unchanged body function was reported in various tissues including brain, kidney, lung, and intestine. No abnormalities were linked to cardiovascular functions such as heart rate, blood pressure, and blood chemistry. Nevertheless, loss of β-arrestin 1 in the heart induced higher cardiac performance as measured by ejection fraction in response to isoproterenol, a βAR agonist, suggesting its role on β-adrenergic receptors [124]. Homologous recombination was used to inactivate the gene encoding for β-arrestin 2 [122]. Similarly to β-arrestin 1 knockout mice, loss of β-arrestin 2 in mice did not impact viability nor organ morphology. Notably, the analgesic effect of morphine was significantly enhanced in the knockout animals due to μ-opioid receptor signaling [125].

## 4. GRK2 and β arrestin in diseases

### 4.1. In cardiovascular and metabolic pathology

GRK2 and GRK5 are the prominent GRKs in the heart and elevated in heart disease [126]. GRK2 is paramount for both normal physiological cardiac function and disease progression. Upregulation of cardiac GRK2 has been reported in human HF [127,128] and diabetes [129]. In its canonical role, the accumulation of cardiac GRK2 internalizes βARs, leading to βAR insensitivity. This is particularly important to cardiac physiology as βARs are key to chronotropic and ionotropic regulation of the cardiovascular system in response to norepinephrine [130]. Increase in circulating catecholamines and upregulation in cardiac GRK2 are often observed in HF patients, both of which promote βAR down-regulation, cardiac hypertrophy, and myocyte apoptosis [128]. Notably, in response to chronic isoproterenol stimulation, β-arrestin-mediated EGFR transactivation, independent of G-protein activation, confered cardioprotection when compared to control animals [131]. Thus, drugs that selectively maintain cytoprotective mechanisms could be beneficial to HF patients.

Mechanistically, studies involving GRK2 in cardiovascular and metabolic pathologies have mainly stemmed from genetic mouse models. Although GRK2 deletion in mice is embryonically lethal, heterozygous GRK2 knockout mice have been used to investigate the role of GRK2 in HF progression in response to β-adrenergic stimulation [132]. Indeed, isoproterenol stimulated inotropic activity was more sensitive in cardiac-specific GRK2 knockout mice, where it restored inotropic and lusitropic responsiveness in acute isoproterenol-induced tachyphylaxis but led to accumulated catecholamine toxicity [115]. Studies have also found that cardiac-specific GRK2 ablation protected the heart from myocardial infarction (MI), reversed pathological left ventricular remodeling [116], and enhanced Ca^2+^ handling while diminishing tissue remodeling [133]. Circulating catecholamines are regulated by adrenal GRK2 which has been implicated in the pathophysiological features of HF, in particular regulating cardiac rate and contractility [134]. Studies have found that adrenal gland-specific inhibition of GRK2 decreased the level of plasma catecholamines and upregulated cardiac βAR signaling, improving cardiac function in HF [135]. Overall preventing GRK2 upregulation in the heart appears to be a viable strategy to treat HF. Notably, recent studies have shown that whole pancreas GRK2 knockdown leads to decreased glucose-mediated insulin secretion, increased weight gain when in a high fat regimen, and decreased cardiac function [123]. Kidney inhibition of GRK2 has also been reported to negatively impact kidney function and blood pressure [136]. GRK2 accumulation in the heart turns-off insulin signaling and inhibits glucose uptake [137]. Noteworthy are the studies showing that high-fat diet (HFD) regimen in cardiac-specific GRK2 inhibitor peptide mice elicited an obesogenic phenotype [138]. Key to untangling these confounding phenotypes are future studies detailing the link between GRK2 and metabolism. Overall, it is important that pre-clinical studies aimed at GRK2 inhibition for HF consider the impact in other organs and how dietary regimens may overall lead to physiological alterations.

Both β-arrestin 1 and 2 are abundantly expressed in cardiomyocytes and contribute to HF progression by enhancing PKA signaling of cardiac βAR [23]. β-arrestins were found to induce transactivation of Epidermal Growth Factor Receptor (EGFR) signaling via β1AR [131]. One of the regulators of blood homeostasis, angiotensin II (Ang II) receptor, plays an important role in enhancing the renin-angiotensin system (RAS) and is involved in the progression of cardiac hypertension and HF [139]. Studies have found that cardiac-specific overexpression of Ang II receptor type 1 (AT1) induces cardiomyocyte apoptosis by activation of Src and translocation of nuclear phospho-ERKs leading to cytoplasmic accumulation of phospho-ERKs [140], and the cytoplasmic accumulation of phospho-ERKs in response to Ang II in cultured cardiac myocytes could be enhanced by the interaction with β-arrestin and mediate a hypertrophic response [141,142]. Cardiomyocytes from heterozygous GRK2 KO mice were also found to have enhanced fractional shortening in response to “biased agonist” of the AT1 receptor (SII) by inducing β-arrestin 2 recruitment [143]. In addition, it has been reported that overexpression of β-arrestins increases mitochondrial superoxide production, while the knockdown decreases ROS production in failing cardiac fibroblasts and human HF [106,144,145]. Metabolically, the heart relies on exogenous glucose and fatty acids, which are tightly controlled by insulin signaling. Insulin resistance plays a key role in the pathogenesis of HF [146]. Studies have found that inhibiting β-arrestin 1 attenuates glucagon-like peptide 1 (GLP1) signaling in cultured pancreatic β cells, resulting in lower cAMP levels, decreased activity of ERK1/2, and CREB, followed by impaired insulin secretion [147]. β-arrestin 1 can alter insulin signaling by inhibiting insulin-induced proteasomal degradation of IRS-1 and the inhibition of beta-arrestin-1 leads to enhanced IRS-1 degradation and accentuated cellular insulin resistance [148]. Similarly, β-arrestin 2-KO mice developed insulin-resistance, followed by decreased insulin-induced phosphorylation of Akt, GSK-3β, and FOXO1 [149]. Conversely, adipocyte-specific β-arrestin 2 knockout mice showed reduced adiposity and striking metabolic improvements when consuming excess calories [150]. In fact, in the adipose tissue β-arrestin 2 is proposed to be a potent negative regulator of β3ARs. As for GRK2 signaling, better understanding specific mechanisms involving β-arrestins in various tissues will guide future treatment strategies and lead to clarifications on the role of arrestins in metabolic regulation.

### 4.2. In Neurodegenerative pathology

Dopaminergic neurotransmission in the central nervous system (CNS) regulates behavioral responses such as locomotor activity and reward responses. The loss of dopaminergic cells participates in the progression of neurodegenerative pathologies like Parkinson’s disease and AD. Several studies have shown evidence that β-arrestins signaling downstream of internalized receptors are important for the regulation of dopamine-dependent behaviors [151,152]. Dopamine (D) receptors are GPCRs that share similarities with βARs [153]. Both D2 and D3 receptors couple to Gαi and are the most abundant receptor in the brain, D2 receptors, undergo rapid GRK phosphorylation and arrestin binding, which leads to receptor internalization [43]. Moreover, using different antipsychotics, the D2 receptor was shown to participate in the formation of β-arrestin-mediated signaling complexes by negatively regulating Akt activity [154,155].

GRK2 is expressed in multiple brain regions, including major dopaminergic regions [30]. D3 receptors are localized in the limbic cortex, striatum, hippocampus, and other brain regions that are associated with schizophrenia [156] and found to affect schizophrenia-like behavior of rats [157]. It is reported that the phosphorylation of the D3 receptor by GRK2 disrupts the interaction between the D3 receptor and filamin A (FLNA), potentially through a direct interaction with β-arrestin 2 and regulation of D3 receptors in lipid rafts [158]. Interestingly, internalization of D3 receptors stimulated by a novel compound SK609, was GRK2-dependent without the involvement of β-arrestin 1 or 2 with a proposed mechanism involving clathrin [159]. Conversely, another study reported agonist-induced receptor internalization dependent on β-arrestin 2 but independent of GRK2-mediated receptor phosphorylation [160]. Thus, how GRK2 and β-arrestins participate in D3 receptor internalization and signaling remains debatable.

GRK2 has also been implicated in AD. In a rat model of AD, GRK2 was upregulated in the cerebrovascular system at an early stage [97], and GRK2 immunoreactivity in AD human samples was significantly increased. The increase in GRK2 was thought to be linked to increased oxidative stress in response to chronic hypoperfusion, resulting in dysregulated cerebrovascular metabolism homeostasis [161]. Thus, GRK2-mediated mitochondrial localization found in AD patients has been proposed to be a marker of brain damage caused by early hypoperfusion. Chronic hypoperfusion causes oxidative stress and detrimentally impacts the brain and cerebrovascular homeostasis and metabolism [97,161]. Whether GRK2 upregulation in the AD brain is causative or consequential remains to be determined.

### 4.3. In pain and inflammation

Multiple immune processes involve GPCR-GRK signaling pathways. Leukocytes are known to express a vast number of GPCRs which are known to tightly regulate immune responses and intercellular interactions [162,163]. Higher expression of GRK2 and GRK5 in neutrophils was found in both LPS-induced sepsis in vitro and in septic patients which induces the phosphorylation of chemotactic receptors including CXCR1 and leads to suppression of neutrophil migration.[164]. Chemokine receptors CCR2B and CXCR4 play an important role in pain and inflammation by promoting chemotaxis via the binding of monocyte chemoattractant proteins and the recruitment of immune cells like macrophages [165,166]. GRK2 interacts with chemokine receptor CCR2B and chemokine CXCR4 receptor to negatively regulate ERK activation [58]. Splenocytes from mice with deficient β-arrestin 2 expression showed impaired CCR4-mediated chemotaxis in both trans-well and trans-endothelial migration tests suggesting a positive role for β-arrestin 2 in mediating the chemotactic responses of T and B lymphocytes [167]. In addition, the ERK pathway can be induced by a variety of inflammatory mediators including LPS, TNFα, interleukin-1 (IL-1), IL-8 and prostaglandin (E2) as extracellular signals, where the overexpression of GRK2 inhibited β-arrestin 2-mediated ERK activation [168]. GRK2 can negatively regulate LPS-induced ERK pathway in macrophages and decrease cytokine production. In fact, myeloid-specific knockdown of GRK2 exaggerates inflammatory cytokine and chemokine production in response to LPS stimulation via the activation of MEK-ERK pathway, and results in organ injury in mice [169], as well as the Akt signaling via inhibited CCL2 [170]. Moreover, LPS-induced cytokine release is increased in GRK2 heterozygous mice through the phosphorylation of p38 at site Thr-123 [171], which suggested a direct interaction between GRK2 and p38. GRK2 expression has also been implicated in microglia and macrophage function, particularly in ischemic brain damage. In fact, GRK2 has been postulated to regulate p38-induced release of TNFα in response to LPS during the hypoxic-ischemic brain damage [172]. Interestingly, a recent study suggested that GRK2 inactivation of p38 MAPK pathway involve microRNA-15a/16 epigenetic regulation of neuropathic pain [173]. Additionally, p38 MAPK signaling can also directly phosphorylate GRK2 and inhibit its translocation to the membrane, thereby preventing the internalization of CCR2, an important receptor signaling pathway in migration and activation of monocytes [174]. Taken together, these studies indicate that GRK2 participates in regulating the MAPK pathway in the immune system.

GRK2 is an important regulator of NF-κB, which is inextricably linked to the inflammatory system via the interaction with IκBα [175] and p105 (another member of IκB family) [169]. β-arrestin 2 has been shown to negatively regulate migration of dendritic cells (DCs), and play a role in inflammatory diseases [176], including asthma [177]. Inflammation can cause hyperalgesia and allodynia. This is due to the increased excitability of peripheral nociceptive sensory nerves caused by the stimulation of inflammatory mediators like peptides, chemokines, cytokines, and neurotransmitters [178]. Inflammatory associated chronic pain has been linked to GRK2 signaling. In neuropathic pain, the level of GRK2 was reduced in the spinal cord in mice [179], underlining the importance of GRK2 in regulating inflammatory hyperalgesia. In fact, knockdown of neuronal GRK2 prolonged prostaglandin E2 (PGE2)- or cAMP- induced hyperalgesia, in an ERK-dependent pathway [180], and either the reduction of GRK2 in microglia/macrophages or in peripheral sensory neurons can both lead to enhanced severity and duration of pain in PGE2 induced pathological conditions [179]. Overall, further detailing GRK2 and β-arrestin mechanism of action are warranted to better understand this signaling pathway and to evaluate the pharmacological potential of targeting these proteins in the treatment of acute inflammatory responses, autoimmune diseases, and pain.

### 4.4. In cancer

Tumor angiogenesis is a hallmark of cancer and plays an essential role in tumor initiation, progression, and metastasis. Growth and migration of tumors include arrestin signaling via different signaling pathways like CXCR4·CXCR7 complex [181] and β2AR signaling [182]. Analysis of clinical samples and in vivo experiments in mice support a key role for GRK2 in regulating angiogenesis in a variety of tumors [183,184]. Chemokine receptor activation is known to promote angiogenesis [185]. Unlike the classic chemokine receptor CXCR4, recruitment of β-arrestin 2 is different with atypical chemokine receptors (ACKRs) such as CXCR7 [186]. Studies have confirmed that CXCR4 activation induced Gαi signaling and recruitment of β-arrestin 2 through GRK2, where it facilitated stimulation of ERK1/2 signaling. Interestingly, activation of CXCR7 activates GRK2 through Gβ1 and subsequent β-arrestin 2, leading to receptor phosphorylation [187,188]. CXCR7, also known as ACKR3, is key in the progression of various tumors including lung, glioma, and breast cancer [198]. Internalization of CXCR7 is thought to modulate the chemokine potential via the internalization of its agonist CXCL12 [199]. CXCR7 recruits both β-arrestin 1 and 2 via interactions with GRK2 [189].

Many biased-GPCR signaling events involved in tumor growth are linked to GRK/β-arrestin pathways regulating cell growth and apoptosis. Somatostatin receptors (SSTRs), commonly found in neuroendocrine tumors [189], were shown to recruit β-arrestin 2 and in turn lead to robust SSTR2 internalization and accumulation in endosomes which may further reduce the differentiation rate in cancer cells [190,191]. Interestingly, differential β-arrestin recruitment was observed in cells expressing SSTR2 or SSTR2/SSTR5. Co-expression of SSTR2 and SSTR5, disrupted binding of β-arrestin to the receptor, delaying SSTR2 internalization, prolonging GPCR signaling [190] and activating other downstream pathways including MAPK [192].

Evidence also suggests that expression of GRK2 and β-arrestins are corelated with pituitary adenomas [193]. GPR54, a GPCR that can be activated by the neuropeptide kisspeptin, regulates placentation and tumor metastases [194]. Following activation, GPR54 stimulate phosphorylation of ERK1/2 and p38 MAPK pathway via β-arrestin 2 [195]. Overexpression of GRK2 enhanced desensitization of GPR54 by directly interacting with the receptor in HEK 293 cells [196]. Thus GPR54, a key regulator of the hypothalamic-pituitary-gonadal axis, is a promising target for developing therapies to treat endocrine-related disorders [194]. Moreover, inhibition of GRK2 enhanced degradation of cancer-relevant insulin-like growth factor-1 receptor (IGF1R) and prevented its interaction with recruited β-arrestin 2 [197], restraining malignant cell growth. As malignant tumor growth rely on increased bioenergetic demand, the impact of GRK2 on IGF1R signaling warrants further inverstigation as it holds pharmacological promise to various cancer types. Cellular metabolism is key to all cancers as the bioenergetic potential will greatly influence the ability of cancerous cells to migrate, proliferate, and metastasize. GRK2 and β-arrestin have been recently implicated in various metabolic regulatory signaling pathways, yet how GRK2 and β-arrestin participate in altering oncogenic metabolic landscape and impact proliferation and metastasis remain largely understudied. Overall, data supports the notion that GRKs, especially GRK2/β-arrestin signaling, also holds a promise for anticancer therapy.

## 5. Conclusions

**Fig. 3.**
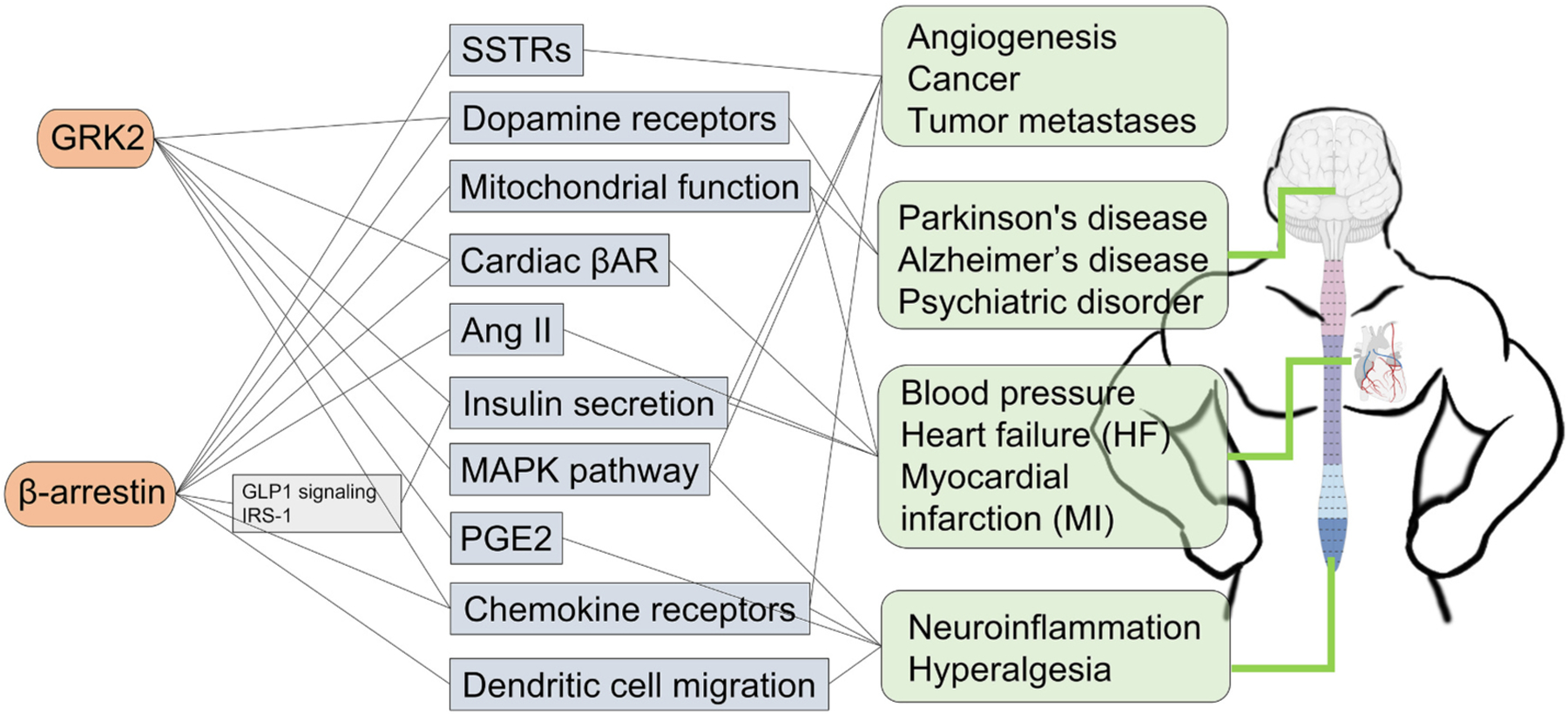
GRK2 and β-arrestin major reported signaling alterations and related pathologies.

GPCR signaling impacts various cell types and cellular functions. It is not surprising that about one-third of FDA-approved drugs target GPCR signaling in one way or another. As there are so many orphaned GPCRs, one can only imagine the pharmacological promise of what still remains unknown. GPCR availability at the plasma membrane is strongly dependent on GRKs’ ability to dynamically phosphorylate these receptors and subsequently recruit arrestins. In addition to the canonical role of these proteins at the plasma membrane, GRK2 and arrestins have been shown to perform a gamut of other non-receptor functions. Focusing on the GRK2/β-arrestin pathway and their link to various diseases (Fig. 3), we reviewed current literature embracing different scientific fields and human diseases. There is a strong consensus that GRK2 and β-arrestins can regulate intracellular signaling proteins in a GPCR-dependent and independent manner. These activities affect various organelles within a cell and exert strong functional and sometimes diverging effects in different organ systems. These observations are particularly important for pre-clinical studies aiming to target GRK2 and/or β-arrestins to treat specific pathologies. It is imperative to delineate further the newly found role of GRK2 and arrestins in metabolism especially as GRK2 emerges as a promising target for HF. Are there unbeknownst outcomes for these potential interventions? Can we design new pharmacological targets that can beneficially target a specific arm of GRK2/β-arrestin signaling? Could we explore this new knowledge to develop personalized medicine strategies? As we untangle this biological “knitted sweater” of signaling network, one thing is certain, the future is bright, exciting, and promising for GRK2 and arrestin pharmacological strategies in the treatment of various human pathologies.

## Figures and Tables

**Table 1:** A subset of GPCRs known to be negatively regulated by GRK2 phosphorylation.

GPCR	Primary Functions	Reference (s)
β2-Adrenergic Receptor	Increases cardiac and skeletal muscle contractility in response to sympathetic stimuli.	[40]
D1-like Dopamine Receptors	Regulate central nervous system reward circuitry and contribute to immune responses [41].	[42]
D2-like Dopamine Receptors		[43]
μ-Opioid Receptor	Key target of natural and pharmacological pain relief.	[44]
Muscarinic Receptors	Broadly expressed receptor family which response to acetylcholine especially from the parasympathetic nervous system.	[45]
CCR9	Regulates immune cell migration particularly for T cells to the GI tract.	[46,47]
CXCR1	Induce leukocyte recruitment and activation at sites of inflammation.	[46]
CXCR2		
CXCR4	Chemokine receptors involved in immune development, hematopoiesis, and vascularization. HIV co-receptor.	[48]
CCR5	Chemokine receptor involved in the migration and activation of leukocytes. HIV co-receptor	[49]
Glucagon like peptide 1 receptor	Incretin hormone involved in regulating insulin secretion, satiety, and cardiac rhythm.	[50,51]
